# Celastrol regulates bone marrow mesenchymal stem cell fate and bone-fat balance in osteoporosis and skeletal aging by inducing PGC-1α signaling

**DOI:** 10.18632/aging.103590

**Published:** 2020-07-28

**Authors:** Li Li, Bing Wang, Yawei Li, Lei Li, Yuliang Dai, Guohua Lv, Pengfei Wu, Pengzhi Li

**Affiliations:** 1 Department of Spine Surgery, The Second Xiangya Hospital, Central South University, Changsha 410011, Hunan, China; 2 Center for Medical Genetics, School of Life Sciences, Central South University, Changsha 410011, Hunan, China

**Keywords:** osteoporosis, Celastrol, BM-MSCs, PGC-1α

## Abstract

Celastrol has recently been identified as a prospective new treatment for obesity and several metabolic complications. However, the effect of Celastrol in osteoporosis (OP) remains unknown. In this study, we demonstrated that Celastrol promotes osteoblast differentiation and prevents adipocyte differentiation in bone marrow mesenchymal stem cells (BM-MSCs) *in vitro*. Mechanistically, Celastrol was able to control the differentiation of BM-MSCs by stimulating PGC-1α signaling. Moreover, administration of Celastrol could alleviate bone loss and bone marrow adipose tissue (MAT) accumulation in ovariectomized (OVX) mice and aged mice. Together, these results recommended that Celastrol could regulate BM-MSCs fate and bone-fat balance in OP and skeletal aging by stimulating PGC-1α, which might act as a possible therapeutic target for OP and for the prevention of skeletal aging.

## INTRODUCTION

As a common metabolic bone disease, osteoporosis (OP) has become a tremendous public health burden on society. Along with existing patterns of OP caused by imbalances between osteoclasts and osteoblasts, recent evidence has suggested that increased accumulation of bone marrow adipose tissue (MAT) occurred at the expense of bone development, which in turn suppressed osteogenic rejuvenation and hematopoiesis [1–4]. Aging or senescent bone marrow mesenchymal stem cells (BM-MSCs) may show a superior tendency of moving toward adipocytes more than osteoblasts [3]. Although, these molecular mechanisms have been fully identified, only a few drugs have been identified for the treatment of osteoporosis, while outcomes produced by these drugs are not satisfactory and are often accompanied by serious side effects. Therefore, it is essential to identify potential therapeutic targets for osteoporosis.

Celastrol is an active ingredient isolated from the outside covering of the root of the conventional Chinese medicinal plant, thunder god vine [5], and has been shown to exert anti-tumor [6], anti-viral [7], antioxidant stress [8], anti-inflammation [9], immunosuppressive [10] and other activities. Previous reports have shown that Celastrol has been extensively utilized to treat rheumatoid arthritis [11], chronic obstructive pulmonary disease [12], systemic lupus erythematosus [13], obesity [14], insulin resistance [15] and nonalcoholic fatty liver disease (NAFLD) [16]. However, the effects of Celastrol on osteoporosis remain undefined.

In this study, we found that Celastrol could regulate BM-MSCs fate and bone-fat balance in OP and skeletal aging by inducing PGC-1α, thereby expanding the spectrum of traditional OP treatment methods available in both experimental and clinical settings.

## RESULTS

### Celastrol promoted the osteogenic differentiation of BM-MSCs *in vitro*

**Figure 1:**
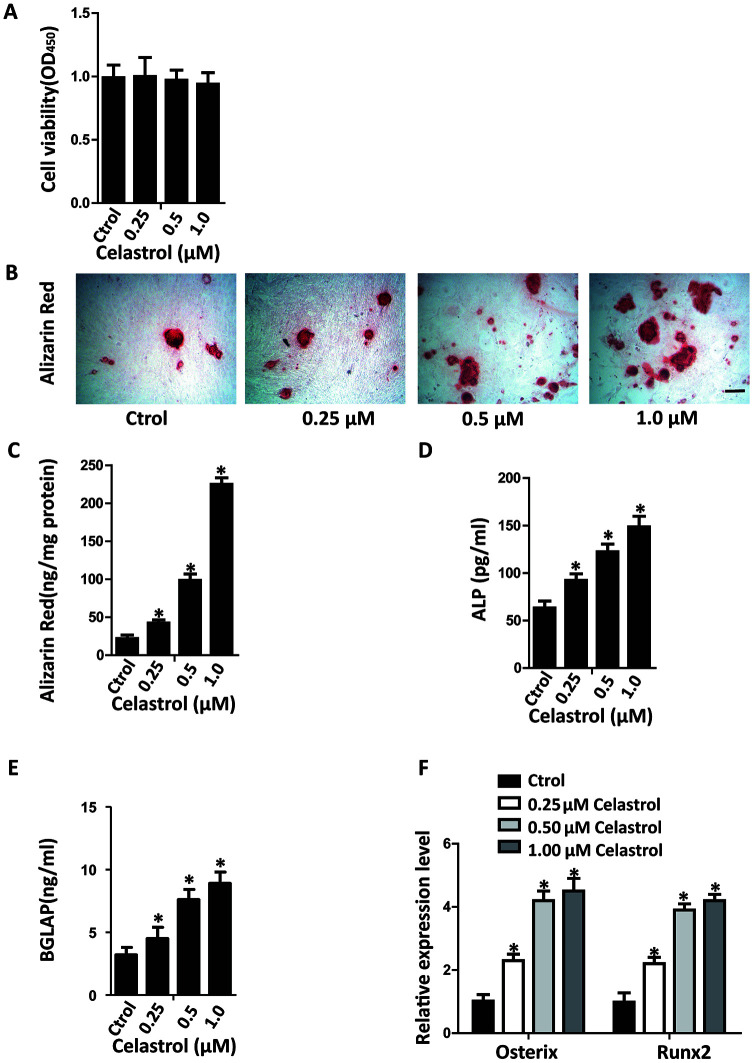
**Celastrol promoted the osteogenic differentiation of BM-MSCs *in vitro*.** (**A**) BM-MSCs were seeded into 96-well plates at a density of 8×10^3^ cells/well and treated with different concentrations of Celastrol for 48 h. Cell viability was determined using CCK-8 assay. (**B**, **C**) Representative images of Alizarin Red S staining (**B**) and quantitative analysis (**C**) of matrix mineralization of BM-MSCs cultured in the osteogenesis induction medium for 21 days. Scale bar: 100 μm. (**D**, **E**) Analysis of ALP activity (**D**) and osteocalcin secretion (**E**) of BM-MSCs cultured in the osteogenesis induction medium for 48 hours (n = 3 per group). (**F**) qRT-PCR analysis of the relative levels of Osterix and Runx2 mRNA expression in BM-MSCs cultured in the osteogenesis induction medium for 48 hours (n = 3 per group). Results are shown as mean ± SD. Statistical significance was determined using analysis of variance (one-way ANOVA). *P < 0.0001 compared with control.

In recent years, research on Celastrol has become increasingly popular due to the therapeutic effects of this active ingredient. In order to investigate the impacts of various dosages of Celastrol on the osteogenic differentiation of BM-MSCs, first a Cell Counting Kit-8 (CCK-8) assay was performed to evaluate the cytotoxicity of Celastrol. The result of CCK-8 assay recommended that Celastrol did not influence cell viability at concentrations of 0.25, 0.5 and 1.0 μM (Figure 1A, *P*=0.8264). Next, the BM-MSCs were cultured in an osteogenesis induction medium with different concentrations of Celastrol. The osteogenic differentiation capacity of BM-MSCs increased in a dosage-dependent manner, as demonstrated using Alizarin Red staining (Figure 1B, 1C, *P*<0.0001 (Figure 1C)). Moreover, osteoblast differentiation markers, alkaline phosphatase (ALP) activity and bone gamma-carboxyglutamic acid-containing protein (BGLAP) secretion also increased, compared with that of control cells (Figure 1D, 1E; *P*<0.000 1 (Figure 1D), *P*<0.0001 (Figure 1E)). Moreover, mRNA levels of the osteoblast transcription factors, Osterix and Runx2, were rapidly elevated due to Celastrol treatment in a dosage-dependent manner (Figure 1F, *P*<0.0001). Together, these results recommended that Celastrol induced the osteogenic differentiation of BM-MSCs *in vitro*.

### Celastrol inhibited the adipogenic differentiation of BM-MSCs *in vitro*

**Figure 2:**
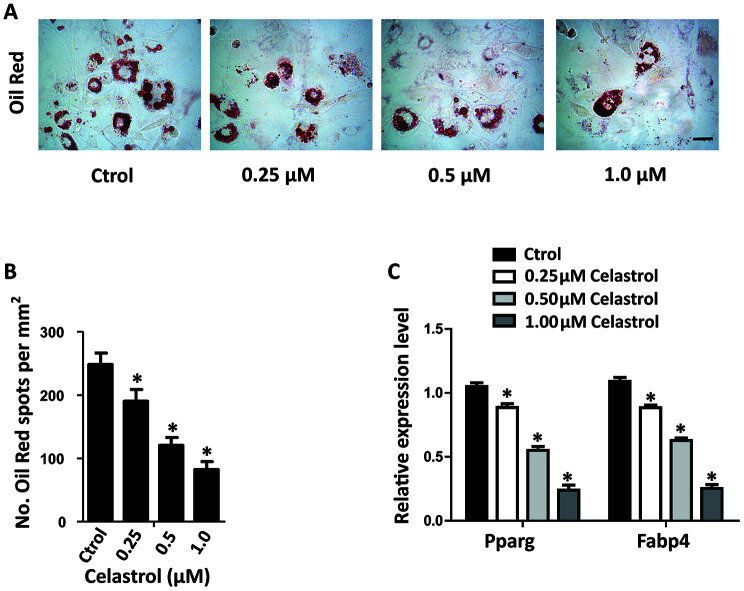
**Celastrol inhibited the adipogenic differentiation of BM-MSCs *in vitro*.** (**A**, **B**) Representative images of Oil Red O staining of lipids (**A**) and quantification of the number of spots (**B**) on BM-MSCs cultured in the adipogenesis induction medium for 14 days. Scale bar: 100 μm. (**C**) qRT-PCR analysis of the relative levels of Pparg and Fabp4 mRNA expression in BM-MSCs cultured in the adipogenesis induction medium for 48 hours (n = 3 per group). Results are shown as mean ± SD. Statistical significance was determined using analysis of variance (one-way ANOVA). *P < 0.0001 compared with control.

In order to investigate the impact of different concentrations of Celastrol on the adipogenic differentiation of BM-MSCs, the cells were cultured in an adipogenic induction medium supplied with different concentrations of Celastrol. The adipogenic differentiation capacity of BM-MSCs diminished in a dosage-dependent manner, as demonstrated using oil red staining (Figure 2A, 2B; *P*<0.0001 (Figure 2B)). Furthermore, the mRNA levels of peroxisome proliferator–activated receptor-g (Pparg) and fatty acid binding protein 4 (Fabp4), two main indicators of adipocyte differentiation, also decreased (Figure 2C, *P*<0.0001). Together, these results recommended that Celastrol inhibited the adipogenic differentiation of BM-MSCs *in vitro*.

### Celastrol regulated the differentiation of BM-MSCs by activating PGC-1α signaling

**Figure 3:**
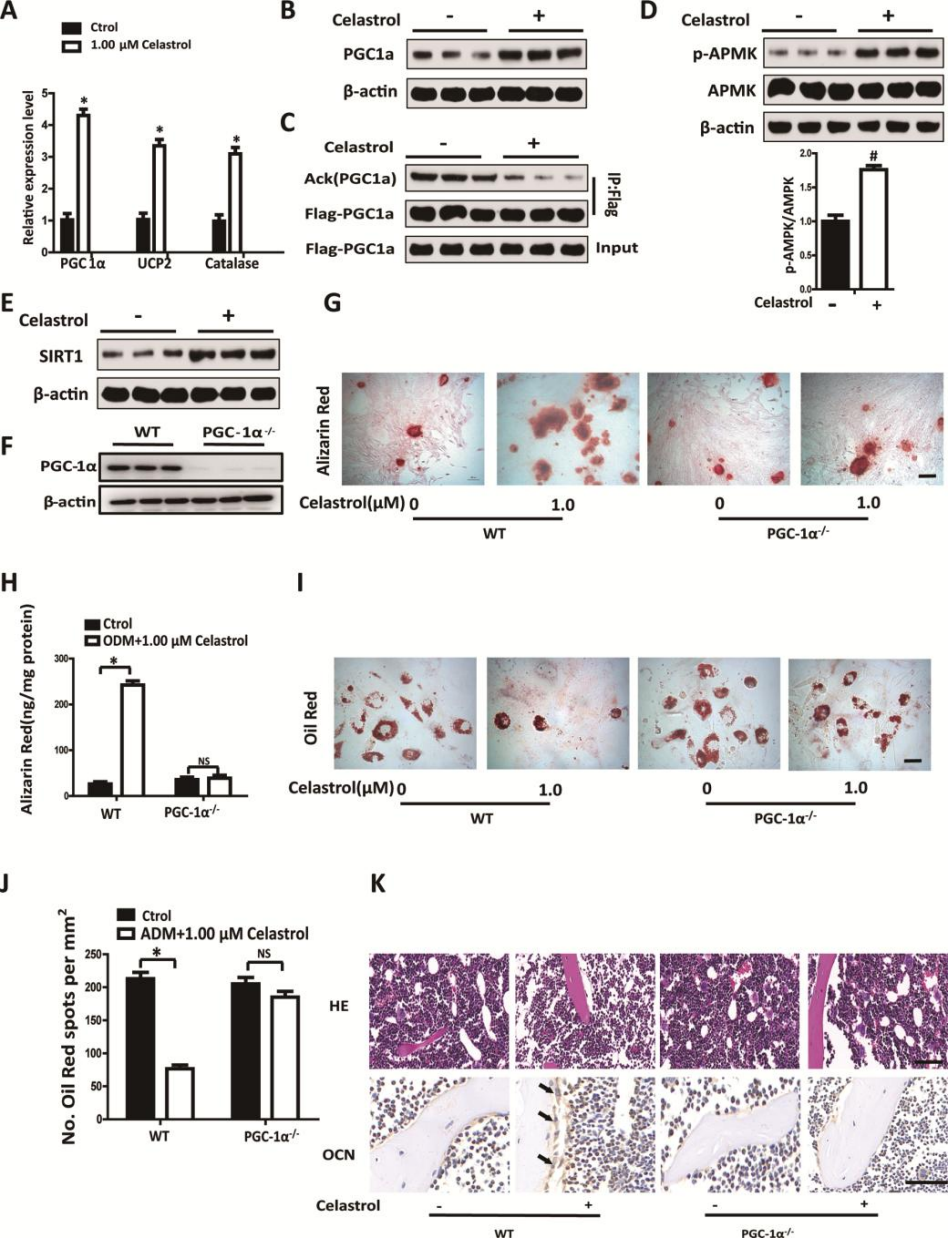
**Celastrol regulated the differentiation of BM-MSCs by activating PGC-1^α^ signaling.** (**A**) Expression levels of PGC-1^α^, UCP2 and Catalase in BM-MSCs treated with the vehicle or Celastrol (1.0 μM, 48 hours) (n = 3 per group). (**B**) Western blotting analysis of the protein levels of PGC-1^α^ of BM-MSCs treated with the vehicle or Celastrol (1.0 μM, 48 hours). (**C**) Western blotting analysis for the detection of PGC1α acetylation levels in PGC1α immunoprecipitates obtained from BM-MSCs transfected with pcDNA-Flag-PGC1α and treated with the vehicle or Celastrol (1.0 μM, 48 hours). (**D**) Western blotting analysis for the detection of pAMPK in BM-MSCs treated with the vehicle or Celastrol (1.0 μM, 48 hours). The levels of pAMPK were quantified using ImageJ software and were normalized to total AMPK levels (**D**, bottom). (**E**) Western blotting analysis of the protein levels of SIRT-1 in BM-MSCs treated with the vehicle or Celastrol (1.0 μM, 48 hours). (**F**) Western blotting analysis of the relative levels of PGC-1^α^ in BM-MSCs transfected with PGC-1^α^ siRNA. (**G**, **H**) Representative images of Alizarin Red staining (**G**) and quantitative analysis (**H**) of matrix mineralization of BM-MSCs cultured in the osteogenesis induction medium for 21 days. Scale bar: 100 μm. (**I**, **J**) Representative images of Oil Red O staining of lipids (**I**) and quantification of the number of spots (**J**) on BM-MSCs cultured in the adipogenesis induction medium for 14 days. Scale bar: 100 μm. (**K**) PGC-1α-knockout (PGC-1α^-/-^) mice (2 month old) and WT mice (2 month old) were ovariectomized. 12 weeks later, they were intraperitoneally injected with Celastrol (200 μg/kg) or DMSO (control) every two days for 4 weeks. H&E staining (top) and osteocalcin immunohistochemical staining (bottom) of the bone were conducted to evaluate the numbers and area covered by adipocytes and osteoblasts after Celastrol treatment. Scale bar: 100 μm. Data are presented as mean ± SD. Statistical significance was determined using the *t*-test. *P < 0.0001; *^#^P* < 0.001 compared with control.

Accumulation of oxidative stress is related to bone loss in OP and skeletal aging [17, 18]. PGC-1α plays an important role in regulating oxidative stress in multifarious mitochondria-rich tissues [19, 20]. More importantly, PGC-1α can reduce ROS in BM-MSCs, participating in controlling lineage decisions between osteoblasts and adipocytes fate of BM-MSCs [21]. We established that the transcript levels of PGC-1α mRNA in BM-MSCs treated with Celastrol were obviously elevated (Figure 3A, *P*<0.0001). Furthermore, levels of UCP2 and Catalase, which are negative regulators of ROS, were also significantly elevated (Figure 3A, *P*<0.0001). Western blotting analysis further confirmed that Celastrol could promote the protein expression levels of PGC-1α (Figure 3B). Moreover, the acetylation levels of PGC1α decreased in the Celastrol-treated group (Figure 3C).

In order to identify pathways involved in activating PGC-1α by Celastrol, we measured the rate of phosphorylation of AMP kinase (AMPK) and expression of SIRT1 in BM-MSCs using western blotting analysis. The results revealed that the Celastrol-treated group showed significantly higher levels of AMPK activity (exhibited as pAMPK/AMPK, *P*=0.0003) and higher levels of SIRT1, compared with the control group (Figure 3D, 3E). In order to confirm whether Celastrol regulated the fate of BM-MSCs via activation of PGC-1α signaling, we silenced PGC-1α signaling in BM-MSCs using siRNAs and then treated BM-MSCs with Celastrol. The successful establishment of the inhibition of PGC-1α in BM-MSCs was confirmed using western blotting analysis (Figure 3F). Interestingly, Celastrol supplementation could enhance osteogenic differentiation and abrogate adipogenic differentiation in BM-MSCs of the WT control group (Figure 3G–3J; *P*<0.0001 (Figure 3H, left panel), *P*<0.0001 (Figure 3J, left panel)). Nevertheless, in the PGC-1α-knockdown group, Celastrol failed to restore homeostasis between osteogenic and adipogenic differentiation of BM-MSCs (Figure 3G–3J; *P*=0.5283 (Figure 3H, right panel), *P*=0.0582 (Figure 3J, right panel)).

In order to further confirm the role of PGC-1a *in vivo*, PGC-1α-knockout (PGC-1α^-/-^) mice (2 month old) and WT mice (2 month old) were ovariectomized. 12 weeks later, the mice were intraperitoneally injected with Celastrol (200 μg/kg) or DMSO (control), every two days for 4 weeks. As expected, Celastrol treatment reduced the number and area of adipocytes in the bone marrow and increased the number and surface of osteoblasts on trabecular and endosteal bone surfaces in WT OVX mice, while the curative effect of Celastrol in PGC-1α^-/-^ OVX mice was offset (Figure 3K). Taken together, these results indicated that Celastrol regulated the differentiation of BM-MSCs by activating PGC-1α signaling.

### Administration of Celastrol alleviated bone loss and MAT accumulation in aged mice

**Figure 4:**
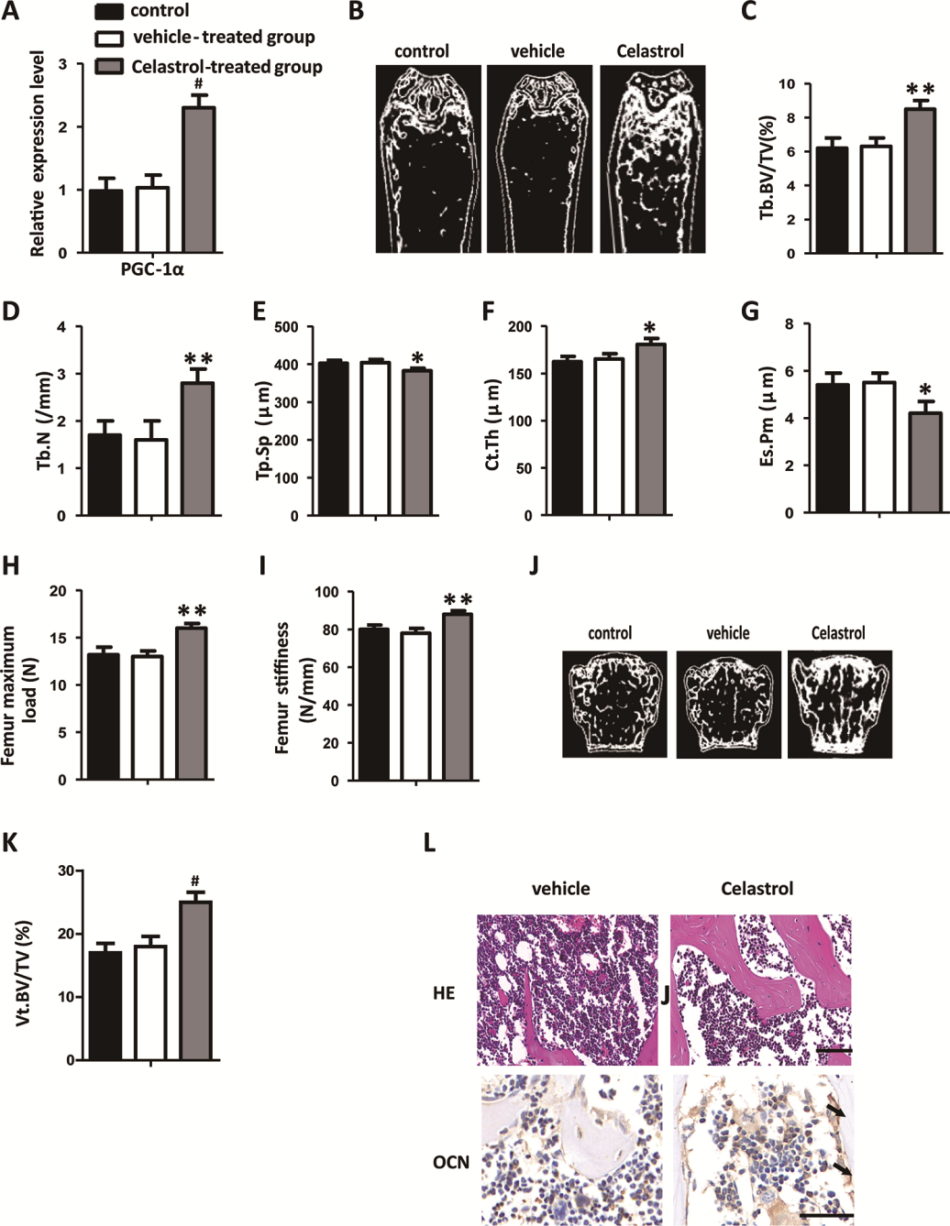
**Administration of Celastrol alleviated bone loss and MAT accumulation in aged mice.** (**A**) mRNA expression level of PGC-1^α^ in the BM-MSCs determined using qRT-PCR (n = 3 per group). (**B**–**G**) Representative μCT images (**B**) and quantitative μCT analysis of trabecular (**C**–**E**) and cortical (**F**, **G**) bone microarchitecture in the femora of Celastrol-treated mice. n = 6-7 per group. (**H**–**I**) Three-point bending measurement of femur maximum load (**H**) and stiffness (**I**). n = 5 per group. (**J**, **K**) Representative μCT images (**J**) and quantification of the ratio of bone volume to tissue volume (**K**) of L4 vertebrae (Vt. BV/TV). n = 6 per group. (**L**) Representative images of H&E staining (**L**, top) and osteocalcin immunohistochemical staining (**L**, bottom). Scale bars: 100 μm. n = 5 per group. Data are presented as mean ± SD. Statistical significance was determined using analysis of variance (one-way ANOVA). *^#^P* < 0.001; **P < 0.01; *P < 0.05.

In order to explore the remedial potential of Celastrol on aging-associated osteoporosis, aged (18 month old) male mice were intraperitoneally injected with Celastrol (200 μg/kg, 98% (HPLC), Sigma, St. Louis, MO) or DMSO (control) every two days for 4 weeks. Mice treated with Celastrol showed increased PGC-1α expression levels, compared with the vehicle-treated group (Figure 4A, *P*=0.0003). As a result, Celastrol augmented trabecular bone volume and number, as well as cortical bone thickness, and reduced trabecular separation and the endosteal perimeter (Figure 4B–4G; *P*=0.0031 (Figure 4C), *P*=0.0084 (Figure 4D), *P*=0.0187 (Figure 4E), *P*=0.0263 (Figure 4F), *P*=0.0259 (Figure 4G)). Moreover, the bone strength of Celastrol treated mice was higher (Figure 4H–4I; *P*=0.0021 (Figure 4H), *P*=0.0031 (Figure 4I)). Vertebral bone volume was also higher in Celastrol treated mice, compared with the vehicle-treated group (Figure 4J–4K; *P*<0.0001 (Figure 4K)). In addition, Celastrol–treated mice showed an obviously lower number and area of adipocytes in bone marrow and a higher number and surface area of osteoblasts on the trabecular and endosteal bone surfaces (Figure 4L). Thus, together these results indicated that the administration of Celastrol alleviated bone loss and MAT accumulation in aged mice.

### Celastrol treatment increased bone formation and decreased bone marrow fat in OVX mice

**Figure 5:**
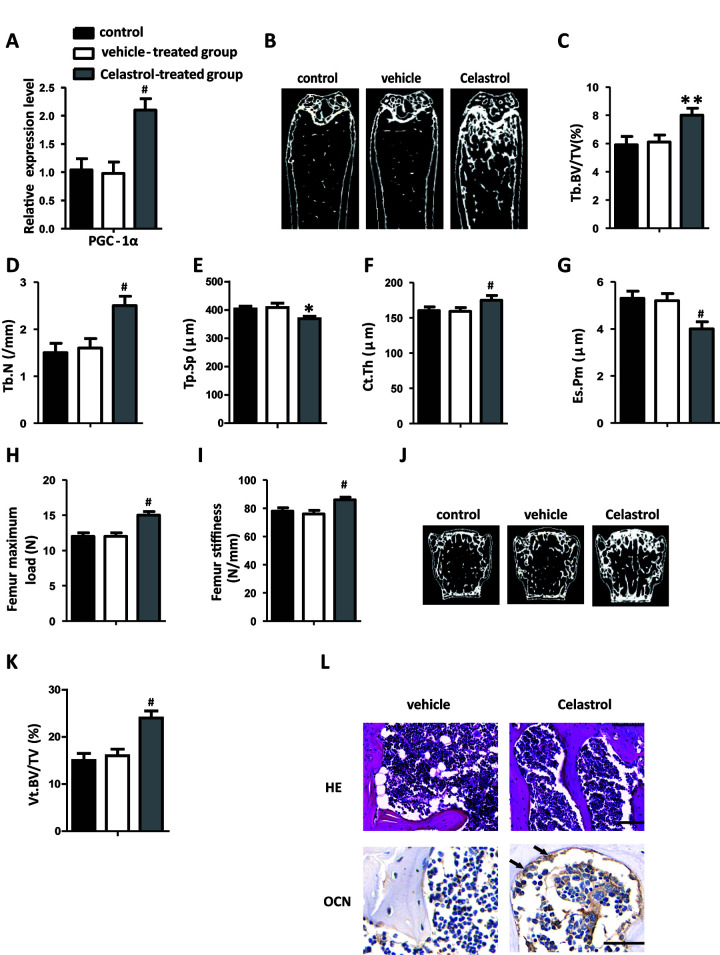
**Celastrol treatment increased bone formation and reduced bone marrow fat in OVX mice.** (**A**) mRNA expression level of PGC-1^α^ in the BM-MSCs determined using qRT-PCR (n = 3 per group). (**B**–**G**) Representative μCT images (**B**) and quantitative μCT analysis of trabecular (**C**–**E**) and cortical (**F**, **G**) bone microarchitecture of the femora of Celastrol-treated mice. n = 6-7 per group. (**H**–**I**) Three-point bending measurement of femur maximum load (**H**) and stiffness (**I**). n = 5 per group. (**J**, **K**) Representative μCT images (**J**) and quantification of the ratio of bone volume to tissue volume (**K**) of L4 vertebrae (Vt. BV/TV). n = 6 per group. (**L**) Representative images of H&E staining (**L**, top) and osteocalcin immunohistochemical staining (**L**, bottom). Scale bars: 100 μm. n = 5 per group. Data are presented as mean ± SD. Statistical significance was determined using analysis of variance (one-way ANOVA). ^#^*P* < 0.001; **P < 0.01, *P < 0.05.

Ovariectomy (OVX) is a well-known model utilized to trigger postmenopausal estrogen deficiency as well as prompt osteoporotic bone loss. In order to further confirm the therapeutic effect of Celastrol, OVX mice were intraperitoneally injected with Celastrol, as mentioned above. Similar to the results obtained from the previous experiment, mice treated with Celastrol showed elevated PGC-1α expression levels (Figure 5A, *P*=0.0008). Furthermore μCT analysis indicated that mice treated with Celastrol showed a significantly greater increase in trabecular bone volume, number as well as cortical thickness, and a reduction in trabecular separation and endosteal perimeter (Figure 5B–5G; *P*=0.0054 (Figure 5C), *P* < 0.0001 (Figure 5D), *P*= 0.0167 (Figure 5E), *P*= 0.0004 (Figure 5F), *P* < 0.0001 (Figure 5G)). Furthermore, bone strength was greater in the Celastrol treated group (Figure 5H–5I; *P*<0.0001 (Figure 5H), *P* < 0.0001 (Figure 5I)). Furthermore, the vertebral bone volume of Celastrol treated mice had increased (Figure 5J–5K; *P* < 0.0001 (Figure 5K)). Meanwhile, adipocyte numbers along with the area of bone marrow covered by them had significantly decreased and the number and surface of osteoblasts on trabecular and endosteal bone surfaces had increased in Celastrol treated mice (Figure 5L). These outcomes recommended that Celastrol treatment increased bone formation and decreased bone marrow fat in OVX mice.

## DISCUSSION

The occurrence of OP along with its complications are rapidly increasing globally. Therefore, it is imperative to identify more effective and safer therapy options for osteoporosis. Previous scientific evidence has found that BM-MSCs have a tendency to differentiate into adipocytes rather than osteoblasts as age increases, resulting in the gradual accumulation of fat and bone loss [22]. Thus, BM-MSCs are regarded as promising therapeutic targets for OP. In this study, our results demonstrated that Celastrol promotes osteoblast differentiation as well as inhibits adipocyte differentiation in BM-MSCs *in vitro*. Consistent with our results, Hong’s research also found that Celastrol exerted an inhibitory effect on lipid accumulation and the adipogenesis of human adipose-derived stem cells (hADSCs) [23]. Additionally, Celastrol could regulate the function of bone marrow-derived endothelial progenitor cells (BM-EPCs) [24].

More importantly, we found that Celastrol controlled the differentiation of BM-MSCs by inducing PGC-1α signaling. Reactive oxygen species (ROS)-induced oxidative stress rises along with aging, resulting in the pathophysiology of aging related OP and postmenopausal osteoporosis [25, 26]. Excessive levels of ROS can prevent the differentiation and development of osteoblasts [27]. PGC-1α performs an imperative function in defending against ROS produced by mitochondrial activity via its capability to stimulate several antioxidant enzymes, including SOD, catalase and glutathione peroxidases [28]. The results of Yu’s research study indicated that PGC-1α is critically involved in determining the fate of BM-MSCs as well as the prevention of MAT buildup as a result of OP and skeletal aging [21]. In our study, we found that mRNA and protein expression level of PGC-1α in BM-MSCs treated with Celastrol were obviously elevated. Furthermore, levels of UCP2 and Catalase, which are negative regulators of ROS, also significantly increased. Likewise, other reports also found that Celastrol could augment PGC-1α expression in adipocytes and skeletal muscles [29, 30].

AMPK and SIRT1 are major upstream regulators of PGC-1α and are inhibited in pathological conditions such as oxidative stress and aging [31]. The activation of AMPK and SIRT1 produces beneficial effects on these conditions. In NAFLD mice, Celastrol could enhance the phosphorylation of AMPK and induce hepatic SIRT1 expression [16]. Consistently, our results revealed that Celastrol could increase AMPK phosphorylation and SIRT1 protein expression levels. Taken together, our data recommended that Celastrol regulated the differentiation of BM-MSCs by activating the AMPK/SIRT1-PGC-1α signaling pathway. Similarly, Wang’s study also found that Celastrol could exert an anti-inflammatory effect in liver fibrosis by increasing AMPK- PGC-1α signaling [32]. In diabetic rats, Celastrol was found to have exerted antioxidant effects on the skeletal muscle, partially by regulating the AMPK-PGC1α-Sirt3 signaling pathway [30].

Celastrol is a traditional Chinese medicine that exerts many biological activities. Celastrol could attenuate intrahepatic cholestasis in pregnancy by preventing matrix Metalloproteinases-2 and -9 [33]. Ma et al. found that Celastrol exerted protective effects against obesity and metabolic dysfunction via stimulation of the HSF1/PGC1α transcriptional axis [34]. It has been documented that Celastrol-induced the prevention of NF-κB scheme associates by exerting an anti-inflammatory response [35, 36] and anti-cancer effect [37]. Furthermore, Celastrol could ameliorate acetaminophen-induced oxidative stress as well as cytotoxicity in HepG2 cells [38]. However, only a few studies have been conducted on the therapeutic effect of Celastrol on osteoporosis. In this study, we established that the administration of Celastrol could alleviate bone loss and MAT accumulation in old mice and OVX mice.

These outcomes indicated that Celastrol could regulate bone marrow stem cell differentiation and bone-fat balance in OP and skeletal aging by stimulating PGC-1α, which might act as a possible therapeutic target for OP and for the prevention of skeletal aging.

## MATERIALS AND METHODS

### Cell culture

Mice BM-MSCs were acquired and cultured, as described in previous reports [39, 40]. In brief, bone marrow cells acquired from the bone marrow cavity of 8 week old mice were incubated at 4°C for 20 min along with the following antibodies: Sca-1, CD29, CD45 and CD11b. Thereafter, Sca-1^+^CD29^+^CD45^−^CD11b^−^ BM-MSCs were separated using flow cytometry (BD Biosciences) and cultured in DMEN supplemented with 10% FBS (Gibco, Invitrogen, USA) and 1% penicillin/streptomycin.

For the transfection of PGC-1α siRNA and its respective negative control (NC), the BM-MSCs were seeded into 12-well plates and transfected on a lipofectamine 2000 system (Thermo Scientific), according to manufacturer's recommendations.

### Cell viability

We used CCK-8 assay to assess the viability of BM-MSCs after Celastrol treatment, as instructed by the supplier. Absorbance was measured at 450 nm via a microplate reader (Thermo Electron Corp).

### Animals

Pathogen free (SPF) C57BL/6J mice were obtained from Hunan SLACCAS Jingda Experimental Animal Co. Ltd., while PGC-1α^-/-^ mice were obtained from Jackson Laboratories. All animals were housed under 12-hour light/dark cycles and were provided unrestricted access to food and water, unless otherwise specified. This study was approved by the Animal Care Committee of Central South University.

For prior to ovarian surgery, OVX mice (2-month-old) were intraperitoneally injected with ketamine (80 mg/kg.bw) plus xylazine (10 mg/kg.bw). Then, the mice were kept in the lateral position and the dorsal and ventral skin was disinfected with a cotton soaked in alcohol. An incision of about 5 mm in length was made on the area ventral to the erector spinae caudal from the last rib through ophthalmology. The lower lumbar muscle was cut to locate the ovaries, which was surrounded by adipose tissue. Both sides of each ovary was ligated and the ovaries were removed. Once the surgery was completed, the incision was sutured, and the mice were placed in warm cages for recovery.

### Administration of Celastrol

For intraperitoneal (i.p.) treatment, mice received 25 μl of vehicle (DMSO) on four consecutive days as acclimation before the doses of Celastrol or vehicle treatment indicated were administered. Celastrol was dissolved in DMSO solution and the mice were administered intraperitoneal injections of Celastrol (200 μg/kg), every two days for 4 weeks. Vehicle groups received the same volume of DMSO for control purposes during the experiments.

### Osteogenic differentiation and mineralization assay

In order to induce osteoblastic differentiation, BM-MSCs were cultured in 24-well plates at appropriate densities in an osteogenesis induction medium for 48 hours. Then, the culture media were obtained for evaluation of ALP activity and osteocalcin levels using ELISA kits, as instructed by the supplier.

In order to induce osteoblastic mineralization, the above mentioned process was performed in 6-well plates at appropriate densities with an osteogenesis induction medium for 21 days. Alizarin Red staining was conducted and used to quantitatively assess cell matrix mineralization.

### Adipogenic differentiation assay

In order to induce adipogenic differentiation, BM-MSCs were cultured in 6-well plates at a density of 2.5 × 10^6^ cells/well in an adipogenesis induction medium for 14 days. Oil Red O staining was performed to identify mature adipocytes in the culture.

### Immunoprecipitation and Western blotting analysis

Cells were transfected with pcDNA-Flag-PGC1α. After 24–48 hours, the cells were lysed in a lysis buffer with a protease inhibitor cocktail, cleared using centrifugation, and subjected to immunoprecipitation using Flag-conjugated beads (Sigma-Aldrich). After 2 to 3 hours, the beads were washed, resuspended in a protein loading buffer, and boiled. Then, the supernatant was subjected to SDS-PAGE and proteins were detected using the indicated antibodies.

### Immunohistochemical staining

Immunohistochemical staining was conducted, as previously described [41, 42]. Bone segments were treated for antigen recovery through assimilation with 0.05% trypsin at 37°C for 15 minutes, and were then probed using a primary antibody against osteocalcin (Takara) overnight at 4°C. Consequently, an HRP-streptavidin recognition system (Dako) was utilized to distinguish immunoactivity, followed by counterstaining with hematoxylin (Sigma). The segments probed with polyclonal rabbit IgG (R&D Systems Inc.) acted as negative controls.

### qRT-PCR analysis

Total RNA was extracted using TRIzol reagent (Thermo Fisher). qPCR was performed using a PrimeScript RT reagent Kit (Takara) and SYBR Green PCR Master Mix (Takara). Each value was adjusted by using β-actin levels as reference. The list of primers used are mentioned in Table 1.

**Table 1:** Primer sequences used for real-time PCR analysis.

**Gene**	**Forward primer**	**Reverse primer**
Actin(mouse)	GGCTGTATTCCCCTCCATCG	CCAGTTGGTAACAATGCCATGT
Actin(human)	CGTGGACATCCGCAAAGA	TCGTCATACTCCTGCTTGCTG
Runx2 (mouse)	ACTTCCTGTGCTCCGTGCTG	TCGTTGAACCTGGCTACTTGG
Osterix (mouse)	ACCAGGTCCAGGCAACAC	GCAAAGTCAGATGGGTAAGTAG
PPAR g (mouse)	GACCACTCGCATTCCTTT	CCACAGACTCGGCACTCA
Fabp4 (mouse)	AAATCACCGCAGACGACA	CACATTCCACCACCAGCT
PGC-1α (mouse)	AGCCGTGACCACTGACAACGAG	GCTGCATGGTTCTGAGTGCTAAG
PGC-1α(human)	TCTGAGTCTGTATGGAGTGACAT	CCAAGTCGTTCACATCTAGTTCA
UCP2 (mouse)	ACAAGACCATTGCACGAGAG	ATGAGGTTGGCTTTCAGGAG
Catalase(mouse)	TGAGAAGCCTAAGAACGCAATTC	TGAGAAGCCTAAGAACGCAATTC

### Micro-CT analysis

The femurs were separated from mice and fixed in 4% paraformaldehyde overnight. Next, they were imaged and evaluated using high-resolution micro-CT analysis (Skyscan 1172, Bruker MicroCT). We selected an area of 5% of femoral length below the growth plate for examination. Trabecular bone volume per tissue volume (Tb. BV/TV), trabecular number (Tb.N), trabecular separation (Tb.Sp) and trabecular thickness (Tb.Th) were determined.

### Statistical analysis

The results are expressed as mean ± SD. Two-tailed Student’s t test (for comparison between two groups) as well as one-way ANOVA (for comparison between multiple groups) were performed. All experiments were repeated a minimum of 3 times. A *P* value of < 0.05 signified statistical significance.
